# Relation between Cortical Activation and Effort during Robot-Mediated Walking in Healthy People: A Functional Near-Infrared Spectroscopy Neuroimaging Study (fNIRS)

**DOI:** 10.3390/s22155542

**Published:** 2022-07-25

**Authors:** Julien Bonnal, Fanny Monnet, Ba-Thien Le, Ophélie Pila, Anne-Gaëlle Grosmaire, Canan Ozsancak, Christophe Duret, Pascal Auzou

**Affiliations:** 1 Service de Neurologie, Centre Hospitalier Regional d’Orleans, 14 Avenue de l’Hôpital, 45100 Orleans, France; julien.bonnal@chr-orleans.fr (J.B.); fanny.monnet@etu.univ-orleans.fr (F.M.); ba-thien.le@chr-orleans.fr (B.-T.L.); canan.ozsancak@chr-orleans.fr (C.O.); pascal.auzou@chr-orleans.fr (P.A.); 2 Institut Denis Poisson, Université d’Orléans Collegium Sciences et Techniques Bâtiment de Mathématiques, Rue de Chartres, B.P. 6759, CEDEX 2, 45067 Orleans, France; 3 Unité de Neurorééducation, Médecine Physique et de Réadaptation, Centre de Rééducation Fonctionnelle Les Trois Soleils, Rue du Château, 77310 Boissise-Le-Roi, France; o.pila@les-trois-soleils.fr (O.P.); ag.grosmaire@les-trois-soleils.fr (A.-G.G.)

**Keywords:** functional near-infrared spectroscopy, cortical activation, lower extremity, exoskeleton, rehabilitation, training modes, physical assistance

## Abstract

Force and effort are important components of a motor task that can impact rehabilitation effectiveness. However, few studies have evaluated the impact of these factors on cortical activation during gait. The purpose of the study was to investigate the relation between cortical activation and effort required during exoskeleton-mediated gait at different levels of physical assistance in healthy individuals. Twenty-four healthy participants walked 10 m with an exoskeleton that provided four levels of assistance: 100%, 50%, 0%, and 25% resistance. Functional near-infrared spectroscopy (fNIRS) was used to measure cerebral flow dynamics with a 20-channel (plus two reference channels) device that covered most cortical motor regions bilaterally. We measured changes in oxyhemoglobin (HbO_2_) and deoxyhemoglobin (HbR). According to HbO_2_ levels, cortical activation only differed slightly between the assisted conditions and rest. In contrast, bilateral and widespread cortical activation occurred during the two unassisted conditions (somatosensory, somatosensory association, primary motor, premotor, and supplementary motor cortices). A similar pattern was seen for HbR levels, with a smaller number of significant channels than for HbO_2_. These results confirmed the hypothesis that there is a relation between cortical activation and level of effort during gait. This finding should help to optimize neurological rehabilitation strategies to drive neuroplasticity.

## 1. Introduction

Gait and balance impairments caused by neurological disorders, such as stroke, affect many people around the world and impact on independent living and quality of life [1]. Gait rehabilitation is therefore highly important for individuals after stroke. Over the last 2 decades, robot-assisted rehabilitation has become an adjunct to usual therapy both for upper limb rehabilitation and gait training, mainly after stroke [2,3]. Innovative mechanical and motorized devices can provide intensive, repetitive, task-specific, and multi-sensory training, features that drive the neuroplastic changes that enhance motor outcomes [4,5]. Recent meta-analyses found that robotic gait rehabilitation combined with physiotherapy enhances recovery after stroke [6,7]. However, little is known about the neural correlates of gait in robot-assisted gait training (RAGT).

Brain imaging studies using positron emission tomography (PET), functional magnetic resonance imaging (fMRI), and electroencephalography (EEG) have advanced knowledge of both spontaneous and rehabilitation induced neuroplastic changes after brain damage [8,9]. However, the application of these techniques to gait is limited because they are highly sensitive to movement artefacts [10]. Functional near-infrared spectroscopy (fNIRS) is a good alternative for the study of cortical activation during gait. A growing number of studies have used fNIRS during gait in younger and older healthy subjects as well as in people with neurological diseases such as Parkinson’s disease and stroke [11,12,13,14,15].

To determine optimal training parameters for individualized RAGT, it is essential to understand how robotic devices interact with the users. Exoskeletons can control various gait variables, such as gait speed and step length. They can also provide different levels of assistance, from full (passive mode) to different levels of partial assistance (active mode) and even resistance to active movement. However, the level of assistance that should be provided to drive neuroplasticity and optimize outcomes has not yet been clearly defined.

The neural coding of force has been studied in animals [16] and humans with PET [17], fMRI [18], fNIRS [19,20,21,22,23], EEG [24], and magnetic stimulation [17]. Electrophysiological studies in non-human primates found a correlation between neuronal discharge rates in multiple regions of the contralateral motor cortex and exerted force amplitude [16]. In humans, fMRI studies confirmed the relation between increasing neuronal activation and increasing level of force in the contralateral primary motor (M1)/somatosensory cortices, supplementary motor area (SMA), and the premotor cortex (PMC) [25]. More recently, studies involving fNIRS confirmed the relation between force produced and activation within the contralateral and ipsilateral cerebral hemispheres [17,19]. 

Most human studies of the relation between force produced and cerebral activation have involved static, isometric tasks. Only a few studies have evaluated dynamic movements [23,26,27]. They showed that the level of force exerted is correlated with cortical changes within the neuronal network in both the contralateral M1 and the anterior cerebellum [26,27]. Only two studies investigated isometric force in the lower limb. The first reported the neural correlates of quadriceps torque control in people with chronic obstructive pulmonary disease [28] and the second analyzed isometric contractions of the ankle dorsiflexors in healthy controls [18]. 

Many of the fNIRS studies of gait involved recording hemodynamic variations in the prefrontal cortex (PFC) with a dual task paradigm. Only a few studies have focused on the motor cortex [13,29,30,31]. A better understanding of the effect of the level of force generated on cortical activation would guide the use of exoskeletons for gait rehabilitation in individuals with neurological conditions.

The aim of this study was to analyze activation within the motor regions of the brain during exoskeleton-mediated gait, at different levels of assistance, in healthy subjects. Four levels were evaluated: full assistance (A100%), partial assistance (A50%), no assistance (A0%), and 25% resistance (A−25%). We hypothesized that the level of brain activation measured with fNIRS would increase with increasing physical effort.

## 2. Materials and Methods

### 2.1. Participants

Participants were recruited from 1 March to 30 March 2022. Inclusion criteria were aged between 18 and 40 years, with no history of neurological, physical, or psychiatric illness. Twenty-four healthy individuals (8 males, 16 females; mean age 28.5 (SD 5.1) years, range 21–39) were included. Two additional individuals were initially recruited, but their data could not be analyzed owing to the poor quality of the fNIRS signal. 

The study was explained to all participants, and they provided written, informed consent prior to participation. The study was approved by Institutional Review Board CPP OUEST II—ANGERS on 12 January 2022 (no: 2021/85) and was registered on Clinical trials (no: NCT05298943).

### 2.2. Protocol

The protocol consisted of walking 10 m with the Atalante^®^ exoskeleton (Wandercraft, Paris, France) in 4 different conditions:

- full assistance (A100%): the participant is passive;
- 50 % assistance (A50%): movement is partially assisted;
- no assistance (A0%): the participant is fully active;
- 25% resistance (A−25%): movement is resisted.

The effects of gravity and inertia of the exoskeleton and the user were calculated in real time by an algorithm. In the full assistance condition (A100%), these terms were compensated. In the no assistance level (A0%), only the inertia and gravity of the exoskeleton were compensated, not those of the user. In the resistance condition (A−25%), artificial terms of inertia and gravity were added. 

The order of conditions was randomized between participants; thus, there were 24 possible orders. Eight trials were recorded for each condition. All participants had a 10 to 15 min period of habituation with the exoskeleton before the recording session. They then stood quietly for 1 min before the trials began. Each 10-m trial lasted around 25 to 35 s. Random jitters were used to vary the durations of the rest periods between trials (from 25 to 35 s) to reduce possible resonance effects. The whole recording session lasted approximately 40 min per participant.

### 2.3. The Robotic Device

**Figure 1:**
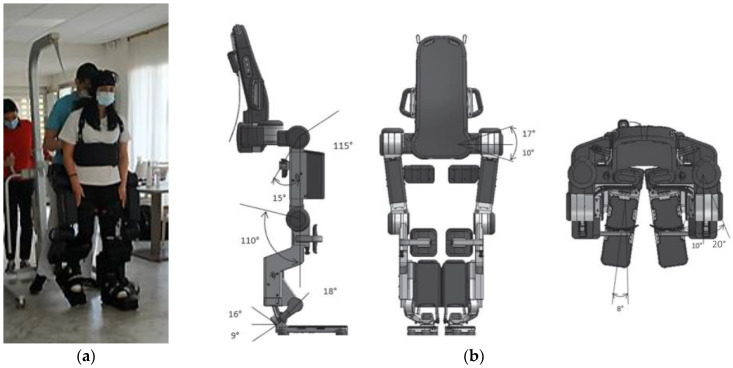
Atalante^®^ exoskeleton (Wandercraft company, Paris, France). (**a**) Participant using the exoskeleton Atalante^®^; (**b**) mechanical design with ranges of motion.

The exoskeleton, Atalante^®^, was developed by the French startup company Wandercraft. The exoskeleton system (Figure 1) is a lower-body exoskeleton with 12 actuated joints [32]: 3 actuated joints control the 3 planes of hip motion, a single actuated joint controls knee flexion/extension, and 2 actuated joints control ankle inversion/eversion and dorsiflexion/plantarflexion. 

The joints that control hip and knee motion are each actuated by a brushless DC motor. The ankle joints have a more complex actuation mechanism that provides rotation in the sagittal plane and about the Henke’s axis. The position and velocity of each actuated joint is measured using a digital encoder. Additionally, the exoskeleton has 4 inertial measurement units (IMUs) that are positioned on the torso, pelvis, left shank, and right shank and which provide additional information about the position and orientation of the device in space. To detect ground contact, four 3-axis force sensors are attached to the bottom of each foot. All of the actuator and sensors are controlled by an embedded computer unit running a real-time operating system. Other components of the exoskeleton include secure loops for mounting the exoskeleton to an overhead hoist, buttons to change the operating mode of the exoskeleton, a connection port to connect the exoskeleton to a computer, handles on either side of the exoskeleton for the operator to assist the exoskeleton if needed, thigh and shank harnesses to secure a participant to the exoskeleton, thigh and shank length adjustments to change the dimensions of the exoskeleton to match that of a participant, and a torso harness that a participant wears to secure their torso to the exoskeleton.

Before beginning, the following anthropometric characteristics of the participant were measured and inputted into the system to generate a model of each participant: height, mass, thigh length, and shank length. Thigh length was approximated by the distance from the buttock crease to the upper border of the patella with the person in a seated position. Shank length was approximated by the distance from the femoral condyles to the ground with the person in a seated position. The measured thigh length and shank length were used to adjust the leg lengths of the exoskeleton to match that of the participant. The participant model was then created as follows. First, the total height of the participant was used to extrapolate the length of each segment of the participant model. The segments were chosen to be: head, arms and trunk; pelvis; left thigh; left shank; left foot; right thigh; right shank; right foot. Using these segment lengths, the center of mass (COM) and inertial for each body segment were then calculated using anthropometric data. The inertia and COM of each segment is given with respect to the proximal end of that segment. The inertia and COM of each segment are then combined with those of the corresponding segments of the rigid body exoskeleton model to form the participant–exoskeleton system.

### 2.4. fNIRS Data Acquisition

**Figure 2:**
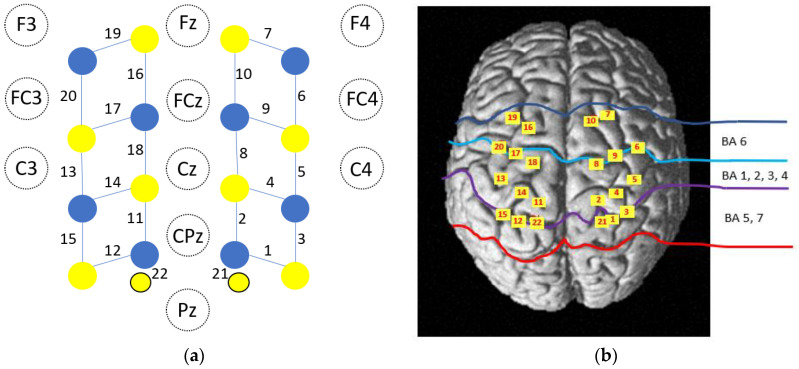
Position of the optodes. Schematics of the optode locations among the EEG 10/20 system. (**a**) A total of 18 optodes, including 10 light source (in yellow) and 8 detectors (in blue), were arranged on the scalp to enable 20-channel measurement. There were two additional short channels (CH21 and CH22). (**b**) The anatomical locations of the optodes were superimposed onto the normalized brain surface in the MNI standard brain template.

Changes in concentration of oxyhemoglobin (HbO_2_) and deoxyhemoglobin (HbR) in the cerebral cortex were measured by a continuous wave optical system: Brite MKII (Artinis Medical Systems, Elst, The Netherlands). The system generates 2 wavelengths of near-infrared light at 760 and 840 nm, which are sampled at a rate of 50 Hz. A total of 10 light sources and 8 detectors lead to 20 standard channels with an inter-optode distance of 3 cm and 2 additional short separation channels (channels 21 and 22) with an inter-optode distance of 8 mm (Figure 2a). Short separation channels were used to remove hemodynamic changes in superficial tissue layers. A 3D digitizer (FASTRACK, Polhemus, Colchester, VT, USA) was used to localize the coordinates of each channel in the MNI standard brain [33], and the coordinates were imported to the NIRS SPM toolbox for spatial registration [34] (Figure 2b).

### 2.5. Preprocessing of fNIRS Data

We considered the HbO_2_ and HbR signals as indicators of the hemodynamic response. The processing was as follows:

1. Trials in which the participant stopped at least twice or for more than 5 s were discarded.
2. Identification and exclusion of bad channels: channels were considered as bad and excluded from the analysis if the coefficient of variation ([standard deviation/mean] × 100) of the raw data was >33%. The function hmrPruneChannels was used (SNRthresh = 3).
3. Optical density conversion: raw data were converted into optical density with the hmrIntensity2OD function.
4. Identification of motion artifacts: time sections were considered as containing motion artifacts if the signal for any given active channel changed by more than 30 times the standard deviation or by more than 5 during a 0.5 s period. The hmrMotionArtifactByChannel function was used (tMotion = 0.5, tMask = 1, STDEVthresh = 30, AMPthresh = 5).
5. Motion artifact correction: sections marked as motion artifacts were corrected with principal component analysis as movement is the principal source of variance. We used the hmrMotionCorrectPCA function (nSv = 0.8).
6. Filtering periodic noise: respiration, cardiac activity and high frequency noise were attenuated with hmrBandpassFilt (hpf = 0, lpf = 0.1).
7. Concentration conversion: corrected optical density data were converted into relative concentration changes with the modified Beer–Lambert law. The age-dependent differential path length factor (DPF) value was calculated for each participant according to the formula proposed by Scholkman and Wolf [35]. Values of DPF for each wavelength were averaged for the group according to the mean age. They were respectively 6.25 and 5.43 for the 760 and 840 nm wavelengths.
8. Short channel contribution and hemodynamic response function (HRF) estimation: the contribution of short separation channels and estimation of the HRF were removed with a Kalman filter dynamic estimator with the hmrDeconvTB_SS3rd function (t range = [−10, 35], gstd = 1, gms = 1, rhoSD_ssThresh = 1).

### 2.6. Data Analysis

Data analysis was performed with MATLAB. The 8 trials for each participant were averaged for each condition. Mean values were calculated for the rest (from 10 s before, to the beginning of the task) and trial periods (from +2 s to +25 s) for each channel. The mean changes in HbO_2_ and HbR between the rest period and condition for each channel were compared using the Student t test. For each condition, 20 t tests were performed; we employed a Benjamini–Hochberg procedure to control the growth of the false discovery rate (FDR) due to multiple comparisons. Significance was set at *p* < 0.05 (FDR-corrected).

## 3. Results

### 3.1. Number of Valid Trials and Duration of Trials

The number of valid trials (/8) for A100%, A50%, A0% and A−25% were respectively 7.75 (SD 0.61), 8 (0), 7.79 (0.83), and 7.75 (0.74). The number of valid trials did not differ between the 4 conditions (F(1,24) = 0.856, *p* = 0.467, *p*η2 = 0.02711). The mean durations of the trials for A100%, A50%, A0%, and A−25% were respectively 30.27 (3.32), 29.74 (2.85), 30.7 (4.96), and 31.28 (4.76) s. Trial duration did not differ between the 4 tasks (F(1,24) = 0.595, *p* = 0.22, *p*η2 = 0.01992).

### 3.2. Comparisons of the Hemodynamic Response between the Conditions

**Figure 3:**
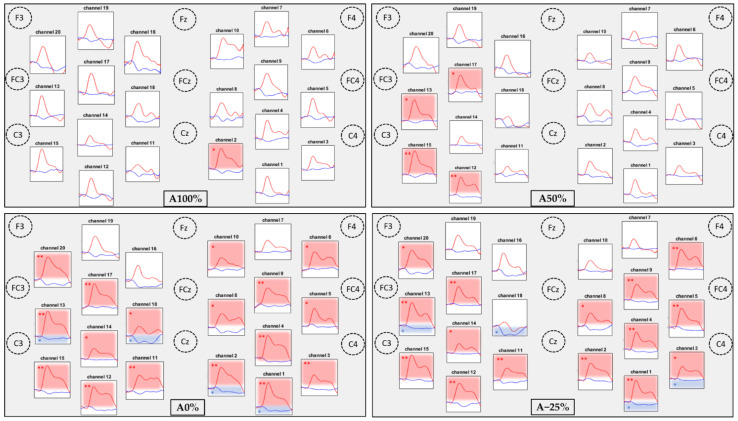
Results of the hemodynamic response by level of assistance (A100%, A50%, A0%, A−25%) for each channel. The results are expressed by mean (average of the participants). Graph locations were organized according to the anatomical correspondence using the EEG 10/20 system. The time window analyzed was 45 s, from 10 s before the beginning of the task to 35 s after the task. The red traces indicate HbO_2_ levels and the blue traces indicate HbR levels. The red boxes indicate a significant difference between rest and task periods for HbO_2_ levels. The blue boxes indicate a significant difference between rest and task periods for HbR levels. * *p* < 0.05; ** *p* < 0.01 (Benjamini–Hochberg correction).

**Figure 4:**
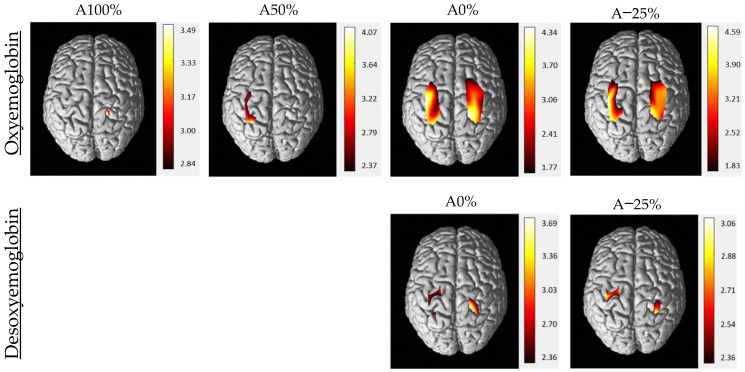
Mean activation maps of cerebral cortex for HbO_2_ and HbR during gait for each level of assistance (A100%, A50%, A0%, A−25%). The data are t values, t: statistical value of sample t-test with a significance level of *p* < 0.05 (Benjamini–Hochberg correction). The change from red to yellow indicates that the degree of activation is from low to high. The coordinates in the figure show the activation range of the cerebral cortex in each level of assistance. Only statistically significant responses were illustrated. The data and maps were calculated and generated by NIRS-SPM.

The hemodynamic responses to the 4 conditions are illustrated by the plotogramms (Figure 3), an NIRS-SPM (statistical parametric mapping for near-infrared spectroscopy) representation (Figure 4), and in Table 1. Overall, the responses are canonical with an increase in HbO_2_ levels and a tendency towards a decrease in HbR levels.

For HbO_2_ levels, only a few significant changes occurred for the 2 assistance conditions (A100% and A50%; see Figure 4). For A100%, only 1 channel in the right primary somatosensory cortex (CH2) showed significant activation. For A50%, 4 channels (CH12, 13, 15, 17) in the left primary somatosensory, somatosensory association, premotor, and supplementary motor cortices showed significant activation (see Figure 3 and Table 1). For the no-assistance and resistance conditions (A0% and A−25%; see Figure 4), bilateral and widespread cortical activation occurred in the primary somatosensory, somatosensory association, primary motor, premotor, and supplementary motor cortices.

For HbR levels, results were similar with no significant difference between the 2 assisted conditions (A100% and A50%), and significant activation of 2 channels in both hemispheres in the unassisted conditions (A0% and A−25%). Fewer channels were active for HbR than for HbO_2_. 

A channel was considered as activated when there was a significant increase in HbO_2_ levels, a significant decrease in HbR, or both. An increase in HbO_2_ associated with a decrease in HbR levels constitutes the canonical response. Significant increases mainly occurred in HbO_2_ levels only or canonical responses. Only channel CH18 in the A−25 condition showed a significant decrease in HbR levels, without any change in HbO_2_ levels.

### 3.3. Comparisons of Task Related Hemodynamic Responses between the 4 Conditions

**Figure 5:**
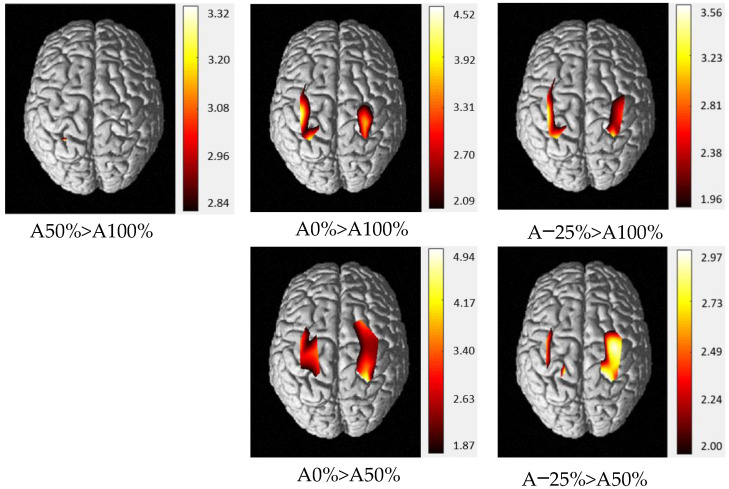
Two-by-two comparisons of HbO_2_ levels for each level of assistance (A100%, A50%, A0%, A−25%). The data are *t* values, *t*: statistical value of sample t-test with a significance level of *p* < 0.05 (Benjamini–Hochberg correction). The change from red to yellow indicates that the degree of difference is from low to high. Only statistically significant comparisons were illustrated. The data and maps were calculated and generated by NIRS-SPM.

Channel activations were compared in pairs for both HbO_2_ and HbR and illustrated only for HbO_2_ levels. According to HbO_2_ levels, two levels of activation could be distinguished (Figure 5). 

The only difference between HbO_2_ levels during A100% and A50% was for a left channel (CH12). No differences were found between A0% and A−25%. However, HbO_2_ levels were higher for many bilateral channels during the 2 unassisted compared with the assisted conditions. The only difference in HbR levels was between A0% and A−25% on CH3 (greater activation for A−25%).

## 4. Discussion

The results of this study in healthy subjects showed that gait with an exoskeleton that required higher levels of effort (i.e., less robot assistance) resulted in more intense and/or more extensive activation of the cortical motor regions, consistent with our initial hypothesis.

### 4.1. Neuronal Activation Is Increased with Higher Levels of Effort

Analysis of the change in HbO_2_ levels from rest showed that assisted gait was associated with lower levels of brain activation, which were more focal, than unassisted gait. We had hypothesized that brain activity would increase progressively with higher levels of effort; however, the changes in HbO_2_ levels suggested two distinct levels of activation for the assisted (A100% and A50%) and unassisted conditions (A0% and A−25%). In contrast, HbR levels did not differ between conditions (except for one channel between A0% and A−25%). 

The difference in sensitivity between the HbO_2_ and HbR chromophores is consistent with the findings of the majority of fNIRS studies. fNIRS measures HbO_2_ and HbR levels separately. During neurovascular coupling, the amount of oxygen supplied is typically greater than that consumed locally, resulting in a substantial increase in HbO_2_ and a slight reduction in HbR in the region. HbO_2_ appears to be more sensitive to brain activation [36,37,38,39,40] but is also more sensitive to artefacts. The HbR response has been found to be more spatially localized (i.e., stimulus evoked HbR level only decreases in a few channels), whereas the HbO_2_ response is more generalized, with responses observed in a higher number of channels [41,42,43]. Analysis and reporting of all the available hemoglobin data in fNIRS are recommended to fully understand condition-evoked cortical activation patterns [44].

We found a non-monotonous increase in cortical activation with reducing levels of assistance. HbO_2_ and HbR levels differed little between the two assistance conditions. Theoretically, in the A100% condition, the participant is entirely carried by the exoskeleton and makes no effort. Although we did not directly measure muscle effort or participants’ perceptions of effort, we consider it unlikely that they were completely passive, which could explain the lack of difference between these two conditions. A study that compared passive and active modes of robotic gait found that muscle activity in the passive mode was about 50% of that recorded during the active mode [45]. Similarly to our results, brain activation levels did not differ between the two lowest effort conditions. In the present study, some participants reported generating more effort in the condition against resistance; however, we did not systematically collect such comments. The lack of difference between the assistance conditions could also be explained by the fact that it was not possible to analyze prefrontal cortex activity owing to the position of the optodes. More marked differences have been found in the anterior SMA and PFC than the posterior motor regions [46]. Indeed, during challenging gait tasks, the SMA, PMC, and PFC are activated [47,48]. 

Several fNIRS and EEG studies evaluated cortical responses during exoskeleton-mediated gait in healthy subjects. The results are partly contradictory because the studies differ considerably in terms of the type of robotic device, the use of EEG vs. fNIRS, study design, types of participants, and parameters used [15,24,45,49]. 

The results of the present study agree with several other studies in healthy individuals. A study of EEG spectral patterns during active and passive robot-assisted gait found significantly suppressed mu (8–12 Hz) and beta (18–21 Hz) rhythms in central midline areas during active gait [24]. This is consistent with our fNIRS results that showed different hemodynamic responses during passive (A100%) and active (A0%) exoskeleton-mediated gait. A comparison of hemodynamic changes (with fNIRS) in the cortical locomotor network area during different conditions (overground stepping, treadmill and RAGT with 100% assistance) and speeds found higher global locomotor network activation during RAGT than stepping and treadmill walking, suggesting that exoskeleton-mediated gait leads to higher levels of brain activation, even with low levels of effort [15]. Walking with an exoskeleton might increase cortical activation in healthy individuals simply because it is somewhat uncomfortable. The same study also found a significant relation between gait speed and the degree of brain activation during both treadmill and exoskeleton gait. The increase in speed could signify a higher level of effort generated by the subject. In our study, gait speed was low, around 1.2 km/h, and comparable in each condition; thus, we believe that the difference between the conditions is unlikely the result of speed variations. 

The results of other studies are, however, in contradiction with our findings. A comparison of HbO_2_ and HbR levels during passive (100% assistance) and active (0% assistance) gait conditions with fNIRS of the bilateral frontal and parietal lobes showed that the passive condition was mainly associated with an increased activation of the parietal cortices bilaterally [45]. 

A comparison of EEG activity during unassisted treadmill gait and robot-assisted treadmill gait at 30%, 60%, and 100% assistance found higher levels of activation in the right primary sensory cortex during the unassisted than the robot-assisted gait [49]. However, the differences in activation between the different levels of robot-assistance were very small. That study is interesting because it evaluated three levels of assistance, which is similar to our protocol. We did not find a difference in brain activation between 100% and 50% assistance. This suggests that changes in effort level must be large enough to drive neuroplasticity, a finding that is important for rehabilitation.

These studies all demonstrate the feasibility and utility of using EEG or fNIRS to increase our understanding of brain activation during gait with an exoskeleton in both physiological and clinical studies. These techniques can be used to evaluate the response to various variables such as gait speed and effort. Both fNIRS and EEG have limitations: fused EEG-fNIRS could help to identify more features that are correlated with brain activation and brain connectivity in the complex process of gait.

Our results showed a relation between the effort generated by the participants and the level of cerebral activation in the regions involved in motor control. Numerous neurophysiological studies have investigated the relation between cortical activity and force. In animal studies, single-cell recordings from the primary motor cortex have shown a direct relation between discharge rates in cortical neurons and static [50] and dynamic [51] force. Force is increased by a combination of well-established mechanisms that involve an increase in the firing rate of already active motoneurons and the recruitment of additional, higher threshold motoneurons. During the control of fine-graded isometric force for a precision grip, the activation of corticospinal and even corticomotoneuronal cells in M1 increases monotonically; however, the firing rate of some cells reduces with increasing force [52]. The relation between neuronal firing and force as well as the rate of force change can be linear, sigmoid, or even logarithmic [53]. 

In humans, various studies have also shown a relation between strength and the degree of brain activation [19,20,21]. However, these studies mostly evaluated upper limb movement and isometric contractions. The cortical changes associated with increased levels of exerted force reflect increases in the central drive to the motoneuron pools of the prime mover muscles [26,54].

### 4.2. Feasibility of fNIRS to Explore Brain Activity during Gait in a Rehabilitation Setting

Knowledge of brain activation patterns during motor activities can guide studies of normal motor control and recovery mechanisms following brain injury as well as help to develop novel rehabilitation strategies in individuals after brain injury. Brain imaging studies have provided a wealth of knowledge regarding motor control. fMRI has very good anatomical precision but is sensitive to motion artifacts; therefore, it has been little used for the study of gait, proximal motor skills, or tasks in ecological conditions [55]. fNIRS is less sensitive to motion artifacts and therefore constitutes a promising option to improve knowledge of brain activation during gait [56]. The field of neurorehabilitation has changed considerably over the past decades, including the development of innovative approaches and technological devices; in parallel, tools that can measure brain activity have emerged to evaluate rehabilitation approaches. These tools must be useable in the rehabilitation setting as well as be reliable, compatible with electronic devices, and usable by therapists or clinical engineers. fNIRS appears to meet these requirements.

fNIRS can be used to record changes in activity in the motor regions of the cortex during gait tasks. To date, most studies of gait using fNIRS have focused on activity of the prefrontal cortex in dual task paradigms, and only a few have considered the motor areas [13,14,15,46]. Our study demonstrated the feasibility of using fNIRS to study changes in cerebral activity in ecological conditions, such as exoskeleton-mediated gait. 

Although our study did not evaluate natural gait, the results showed activation of the sensorimotor regions, close to the median line, during gait as has been previously reported [13]. The gait pattern is generated by spinal interneuronal networks within a so-called central pattern generator [57]. However, locomotion requires cooperation between the spinal networks and descending signals from the brainstem and cerebral cortex. Automatic gait is controlled by the M1, cerebellum, and spinal cord. This pathway is activated during less challenging walking situations (e.g., constant speed, flat ground) in individuals with no neurological pathology. When automatic gait is not possible (e.g., during more challenging tasks) or in the case of pathology, executive control, which involves the SMA, the PMC, the PFC, and basal ganglia, is activated [58].

### 4.3. Implications for Robot Mediated Gait Training in the Rehabilitation Setting

In recent years, robot-assisted rehabilitation has been increasingly integrated into conventional rehabilitation. It offers the possibility to provide early, intensive, task-specific training with multi-sensory stimulation, all of which are considered essential to promote neuroplasticity [59,60]. According to the literature, robotic rehabilitation improves gait capacity, gait speed [61], lower limb muscle strength, step length, and gait symmetry in individuals with neurological disorders [62]. However, the neurophysiological correlates of robot-assisted rehabilitation remain little known. Assessment of the effects of human-robot interaction at the cortical level is important because motor intentions and high-level adaptations of motor patterns for gait are generated in the supraspinal areas. The optimization of rehabilitation for both upper limb paresis and gait impairment requires knowledge of the treatment modalities that enhance neuroplasticity. The repetition of a large number of movements is essential to drive brain plasticity. Furthermore, the movement must be active and the provision of assistance may reduce neuronal activity [63]. Therefore, innovative devices that facilitate movement repetition by assisting the movement must be used with caution.

### 4.4. Limitations

This study has several limitations. First, the level of effort was based on the level of assistance provided by the exoskeleton; we did not use EMG to determine the level of muscle activation. This limitation is common to many other studies that have studied the relation between force control and brain activation [15,25,64,65]. Future studies of gait could consider the relation between EMG activity and brain activity. In the future, it would be interesting to compare brain activation during the different walking phases using the IMU to obtain the different kinematic parameters of walking [66]. Currently, the temporal resolution of the fNIRS device does not allow this analysis which, on the other hand, is possible by using EEG [67]. Second, from a topographical point of view, our study was essentially limited to the medial part of the motor cortex. Some studies have shown more lateral or more anterior activations during gait [13,15,46]. We were limited by the number of optodes that could be used, and only studied the regions that have been shown to be most involved in controlling lower limb movement [24].

## 5. Conclusions

The present work in healthy subjects demonstrated a relation between the level of effort and the level of brain activation during exoskeleton-mediated gait; however, the progression of the activation was non-monotonous. These results extend those of previous studies of isometric contractions to a dynamic motor activity. In the future, this study should be performed in patients with neurological disorders such as stroke to verify that the same relationships between effort and brain activation can be found. Overall, the study also confirmed that fNIRS is a valuable tool to analyze brain function during real life conditions such as rehabilitation sessions. Use of this tool could improve understanding of the cerebral mechanisms that underpin rehabilitation effectiveness.

## Figures and Tables

**Table 1:** Changes in Hb0_2_ and HbR levels between rest and task for each condition. Brain regions over which the channels were located and the corresponding *p*-values (indicating change from rest to the task) for oxyhemoglobin (HbO_2_) and deoxyhemoglobin (HbR) levels for each condition.

Brodmann Areas		*p*-Values—HbO_2_				*p*-Values—HbR			
		A100%	A50%	A0%	A−25%	A100%	A50%	A0%	A−25%
CH1	5—Somatosensory Association Cortex	0.0855	0.0367	0.0005 **	0.0005 **	0.0457	0.0123	0.0027 *	0.0031 *
	7—Somatosensory Association Cortex								
CH2	3—Primary Somatosensory Cortex	0.0010 *	0.0533	<0.0001 **	0.0001 **	0.3184	0.0828	0.0013 *	0.4294
	4—Primary Motor Cortex								
CH3	5—Somatosensory Association Cortex	0.0224	0.0213	0.0049 **	0.0060 *	0.7518	0.0239	0.0295	0.0069 *
CH4	3—Primary Somatosensory Cortex	0.0294	0.1452	0.0002 **	0.0006 **	0.2347	0.0195	0.0161	0.0122
	4—Primary Motor Cortex								
CH5	4—Primary Motor Cortex	0.1508	0.1908	0.0240 *	0.0010 **	0.1172	0.4139	0.3658	0.0425
CH6	6—Pre-Motor and Supplementary Motor Cortex	0.1633	0.0879	0.0090 *	0.0008 **	0.1425	0.7326	0.7273	0.5253
CH7	6—Pre-Motor and Supplementary Motor Cortex	0.0979	0.5044	0.0450	0.2760	0.4147	0.5180	0.4229	0.3234
CH8	6—Pre-Motor and Supplementary Motor Cortex	0.2277	0.0476	0.0086 *	0.0298 *	0.1868	0.0490	0.0811	0.0929
CH9	6—Pre-Motor and Supplementary Motor Cortex	0.1042	0.0158	0.0021 **	0.0012**	0.0339	0.3884	0.3270	0.1213
CH10	6—Pre-Motor and Supplementary Motor Cortex	0.0161	0.2398	0.0276 *	0.1054	0.3731	0.3617	0.3376	0.1917
CH11	3—Primary Somatosensory Cortex	0.2638	0.0517	0.0005 **	0.0028 **	0.0264	0.3252	0.3252	0.0316
	5—Somatosensory Association Cortex								
CH12	5—Somatosensory Association Cortex	0.1746	0.0002 **	0.0002 **	0.0003 **	0.1537	0.0637	0.0155	0.0507
	7—Somatosensory Association Cortex								
CH13	3—Primary Somatosensory Cortex	0.1763	0.0018 *	<0.0001 **	<0.0001 **	0.1620	0.1957	0.0043 *	0.0041 *
	4—Primary Motor Cortex								
CH14	3—Primary Somatosensory and Motor Cortex	0.2355	0.0558	0.0155 *	0.0187 *	0.1696	0.2274	0.0633	0.0751
	4—Primary Motor Cortex								
CH15	5—Somatosensory Association Cortex	0.0508	0.0007 **	0.0003 **	<0.0001 **	0.4901	0.1842	0.0237	0.2276
CH16	6—Pre-Motor and Supplementary Motor Cortex	0.1495	0.3151	0.1059	0.1241	0.0603	0.1517	0.0654	0.1247
CH17	6—Pre-Motor and Supplementary Motor Cortex	0.1271	0.0042 *	0.0003 **	0.0005 **	0.3615	0.6941	0.4628	0.2602
CH18	6—Pre-Motor and Supplementary Motor Cortex	0.3579	0.8857	0.0226 *	0.6183	0.0181	0.0026	0.0049 *	0.0061 *
CH19	6—Pre-Motor and Supplementary Motor Cortex	0.4030	0.1868	0.0625	0.0425	0.2159	0.1225	0.0389	0.1271
CH20	6—Pre-Motor and Supplementary Motor Cortex	0.5002	0.0396	0.0004 **	0.0057 *	0.0604	0.0386	0.0190	0.0166

Significant difference between rest and task-related hemodynamic responses for the different level of assistance (A100%, A50%, A0%, A−25%). *p*-values are absolute. Significance levels were corrected for multiple comparisons using the Benjamini–Hochberg procedure: * *p* < 0.05; ** *p* < 0.01. Channel 21 and 22 are short separation channels and therefore were not listed.

## Data Availability

All data are available in electronic format at the Service de Neurologie, Centre Hospitalier Regional d’Orléans.

## Abbreviations

fNIRS	Functional near-infrared spectroscopy
HbO_2_	Oxyhemoglobin
HbR	Deoxyhemoglobin
RAGT	Robot-assisted gait training
PET	Positron emission tomography
fMRI	Functional magnetic resonance imaging
EEG	Electroencephalography
SMA	Supplementary motor area
M1	Primary motor cortex
PMC	Premotor cortex
PFC	Prefrontal cortex
IMU	Inertial measurement units
COM	Center of mass
DPF	Differential path length factor
HRF	hemodynamic response function
FDR	False discovery rate
NIRS-SPM	Statistical parametric mapping for near-infrared spectroscopy
